# A Randomized Controlled Trial of a Trauma-Informed Support, Skills, and Psychoeducation Intervention for Survivors of Torture and Related Trauma in Kurdistan, Northern Iraq

**DOI:** 10.9745/GHSP-D-16-00017

**Published:** 2016-09-28

**Authors:** Judith Bass, Sarah McIvor Murray, Thikra Ahmed Mohammed, Mary Bunn, William Gorman, Ahmed Mohammed Amin Ahmed, Laura Murray, Paul Bolton

**Affiliations:** a Johns Hopkins Bloomberg School of Public Health, Department of Mental Health, Baltimore, MD, USA; b Heartland Alliance International, Chicago, IL, USA; c University of Chicago, School of Social Service Administration, Chicago, IL, USA; d Trauma Rehabilitation and Training Center; Iraq-Kurdistan Region-Sulaimani and Department of Community Health, Sulaimani Polytechnic University, Technical College of Health, Qirga, Iraq; e Johns Hopkins Bloomberg School of Public Health, Center for Refugee and Disaster Response and Department of International Health, Baltimore, MD, USA

## Abstract

Supportive counseling type interventions are frequently provided to meet the mental health needs of populations in emergency and post-conflicts contexts, but it has seldom been rigorously evaluated. Existing evaluations from low- and middle-income countries provide mixed evidence of effectiveness. While Iraqi Kurdistan experienced relative stability following the fall of Saddam Hussein’s government, the population in the northern Dohuk region has continued to experience periodic violence due to conflicts with neighboring Turkey as well as more recent ISIS-associated violence. We evaluated the impact of a trauma-informed support, skills, and psychoeducation intervention provided by community mental health workers (CMHWs) on depressive symptoms and dysfunction (primary outcomes) as well as post-traumatic stress, traumatic grief, and anxiety symptoms (secondary outcomes). Between June 2009 and June 2010, 295 adults were screened; 209 (71%) met eligibility criteria (trauma exposure and a symptom severity score indicating significant distress and functional impairment, among others) and consented to participate. Of these, 159 were randomized to supportive counseling while 50 were randomized to a waitlist control condition. Comparing average symptom severity scores post-treatment among those in the intervention group with those in the waitlist control group, the supportive counseling program had statistically and clinically significant impacts on the primary outcomes of depression (Cohen’s d, 0.57; *P* = .02) and dysfunction (Cohen’s d, 0.53; *P* = .03) and significant but smaller impacts on anxiety. Although studies by the same research team of psychotherapeutic interventions in other parts of Kurdistan and in southern Iraq found larger effects, this study adds to the global research literature on mental health and psychosocial support and shows that a well-trained and supervised program of trauma-informed support, skills, and psychoeducation that emphasizes the therapeutic relationship can also be effective.

## Abstract (precis)

Providing survivors of torture, imprisonment, and/or military attacks with a counseling program that includes support, skills and psychoeducation by well-trained and supervised community mental health workers can result in moderate yet meaningful improvements in depression and dysfunction.

## INTRODUCTION

Traumatic events, including torture, put people at increased risk for a range of mental health problems.^1^ Physical torture has been shown to be a robust predictor of mental distress in prisoners of war and other conflict-affected or displaced populations years, and even decades, after torture occurred.^1^^–^^3^ To address these problems, the most frequent type of mental health intervention provided in emergency or post-conflict contexts has been basic supportive counseling from trained community workers.^4^ This is consistent with the *Guidelines on Mental Health and Psychosocial Support in Emergency Settings*, from the Inter-Agency Standing Committee (IASC), which recommends that basic supportive counseling components be provided by community health workers to support psychosocial well-being.^5^ The content and activities provided in interventions labeled as basic counseling vary widely. Commonly used strategies for trauma-affected adult populations include problem solving, conflict resolution, psychoeducation (information provided to the participant about their condition), relaxation, and sharing of traumatic experiences, as well as teaching skills in coping, stress management, and basic communication.^6^^–^^14^ While the literature suggests that evidence-based treatments, such as Cognitive Behavioral Therapy, are effective for mental health problems in confict and post-conflict settings (see, for example, Weiss et al. 2016^15^), it is possible that some of the commonly seen depression and anxiety symptoms in such settings may be made more severe due to environmental stress, and these symptoms may be amenable to a more generally supportive counseling approach.

These types of basic supportive counseling programs, as opposed to more structured and manualized pyschotherapies (i.e., therapies that follow a series of prescribed goals and techniques to ensure uniformity across therapists), have seldom been rigorously evaluated,^4^ and existing evaluations from low- and middle-income countries provide mixed evidence of effectiveness for survivors of torture and related traumas. For example, a group problem-solving counseling program delivered in conflict-affected areas of Aceh, Indonesia, showed an effect on improving daily functioning in men and coping in both men and women, but showed no effect on depression or anxiety symptoms.^7^ In a study among torture survivors in Nepal, a multidisciplinary counseling program similarly improved functioning and decreased participants’ somatic symptoms, but did not reduce mental health symptoms.^10^ In a trial in Uganda, the vast majority of Sudanese refugees were found to still meet criteria for post-traumatic stress disorder (PTSD) following a 4-session supportive counseling program that included problem solving, conflict resolution, and psychoeducation components.^8^

Basic supportive counseling programs have seldom been rigorously evaluated.

In contrast, in a treatment trial of former Palestinian political prisoners, participants in an individual counseling program that contained trauma-specific elements, such as desensitization, stress inoculaton, coping skills, and emotion regulation, and that addressed broader social problems saw small but significantly greater improvements in traumatic stress symptoms than waitlist controls.^10^ Female survivors of conflict and associated sexual violence in Liberia participating in a supportive counseling program that included sharing of past traumatic experiences, conflict resolution, and stress management experienced small mean declines in trauma symptoms, while those enrolled in basic skill-building activities and waitlist controls experienced small mean increases in trauma symptoms over the study period.^14^

The population in Iraqi Kurdistan has experienced significant trauma in the past several decades. In the late 1980s, the regime of then-Iraqi President Saddam Hussein conducted a violent campaign in Iraqi Kurdistan, known as the Anfal, that included genocide, chemical weapon attacks, and torture.^16^ Within Kurdistan’s northernmost governorate, Dohuk, hundreds of villages were razed leading up to and during the Anfal in the name of “Arabization.”^16^ While Kurdistan experienced relative stability following the fall of Saddam Hussein’s government, conflict between Kurdish groups and neighboring Turkey continued to produce periodic violence in the Dohuk border region.^17^^,^^18^ Recenly, the brutal advance of ISIS (the Islamic State of Iraq and Syria) has made Dohuk a major site of refuge for displaced Iraqis and refugees.^19^

Even prior to the current ISIS conflict, the population in the Dohuk region of Iraqi Kurdistan has had little access to community-based mental health services for trauma. As part of a larger study to evaluate mental health services for torture and trauma survivors throughout Iraqi Kurdistan, in 2010 we evaluated a counseling program that is based on psychotherapies that are designed for trauma-affected populations and includes specific skills and psychoeducation components to improve the participants’ symptoms of depression, anxiety and trauma, and dysfunction. The intervention was provided by community mental health workers (CMHWs) at Ministry of Health clinics in the Dohuk governorate of Kurdistan, Northern Iraq. The study aimed to assess the impact of the intervention on primary outcomes of depressive symptoms and dysfunction, and secondary outcomes of post-traumatic stress, traumatic grief, and anxiety symptoms. Based on a preliminary qualitative study^20^ conducted with a similar population, depressive symptoms were identified as the most important mental health problem affecting this population, with anxiety, traumatic stress, and traumatic grief as secondary outcomes. Heartland Alliance International (HAI), an international NGO based in the United States with offices and programs in Iraq, developed this intervention as part of a broader program of integrating mental health into the health care system in Iraqi Kurdistan.

## INTERVENTION DESCRIPTION

### Program Development

From 2005 to 2007, HAI implemented an integrated mental health program into the primary health care system in Iraqi Kurdistan, including a comprehensive mental health curriculum for the paraprofessional level, CMHWs. CMHWs were recruited through a joint selection process by the Department of Health in the Dohuk governorate, the Health Staff Association of Kurdistan (a nongovernmental professional group of health staff), and staff of HAI including a study coauthor (AA). The main selection criteria were clinical staff from the local primary clinics who had time and expressed an interest in gaining skills in mental health and psychosocial support and had experience working in rural areas with people who had experienced torture and trauma. The goal was to identify 1 male and 1 female health staff to be trained as CMHWs, although the lack of female staff in the health centers in Dohuk meant that there were more male CMHWs. These staff, who would become the CMHWs, included pharmacists, nurses, and physician assistants, and were permanent employees of the Ministry of Health. None of the CMHWs had any formal mental health training prior to the HAI project.

The community mental health workers recruited for our study included pharmacists, nurses, and physician assistants without any prior formal mental health training.

At the time of the program, there were limited mental health services in the country and those that did exist were concentrated in the major urban areas, with a focus on medication treatment. The HAI program’s first step was establishing a CMHW role within the health centers and providing them with foundational knowledge to deliver care and support. At the time of the current study in 2009, HAI had trained approximately 50 CMHWs in Kurdistan region.

### Training Curriculum

The basic skills curriculum was developed as a 2-week training program that emphasized a social work model of helping and support.^21^ It included information on mental health and illness^22^ and the skills necessary to provide psychosocial support to individuals, with a particular focus on depression, anxiety, and post-traumatic stress. The curriculum emphasized basic knowledge and skills of a helping professional including the therapeutic relationship, compassionate care, maintaining confidentiality, active listening, empathy, and problem solving, as well as core tasks such as medication management, providing psychoeducation, working in the community, and advocacy. In addition to practice evidence derived from working with survivors of torture in the United States,^23^ trauma theory and conceptual frameworks considered important for contexts with historical and ongoing political violence were integrated throughout the curriculum. This included a phase-based orientation to work with survivors of trauma that emphasized the importance of the therapeutic relationship and clinical principles of safety and stability when working with survivors of trauma.^24^ The curriculum used a multisystem or person-in-environment approach^25^ to understanding problems resulting from violence and trauma exposure and a strengths-based orientation to working with clients.^21^ All HAI training included a train-the-trainer manual and a participant workbook. The basic training, together with a series of advanced trainings, in total took place over the course of 2 years and included 30 days (240 hours) of in-person training and monthly field supervision.

The curriculum development team consisted of U.S-based adult learning experts and mental health technical staff as well as Iraqi program staff with diverse expertise in curriculum development, trauma-focused mental health practice and Iraqi culture and society. The project used an iterative, participatory action model for curriculum development, which took several months to complete and included (1) identifying learning needs in collaboration with Iraqi staff and CMHWs; (2) gathering information via interviews with Iraqi staff and CMHWs to map curriculum content; (3) drafting the curriculum; (4) testing the curriculum during pilot train-the-trainer sessions; (5) gathering post-pilot evaluative information to revise training materials; (6) implementing the revised training with CMHWs; and (7) ongoing evaluation and further refinement. This process resulted in locally informed training materials and allowed for ongoing quality improvement. U.S-educated, licensed clinical social workers with expertise working cross-culturally and with torture survivor communities facilitated the train-the-trainer program, while the HAI program staff in Iraq, mainly physicians, facilitated the CMHW trainings.

The project used an iterative, participatory action model to develop the supportive counseling curriculum.

### Adaptation for the Trial

In preparation for this trial, the original basic skills curriculum was adapted into a time-limited trauma-informed support, skills, and psychoeducation intervention so that it could be compared with 2 other trauma-focused manualized interventions that were selected for study. This intervention was designed to include 6–12 sessions for each client depending on particular client needs and to include techniques and skills analogous to what is done at the beginning of phase-oriented treatment for trauma with significant emphasis on the importance of the therapeutic relationship.^24^ This process of adaptation for the study was led by the HAI clinical director (BG), a clinical psychologist with extensive expertise in torture rehabilitation. Together with the Kurdish study psychiatrist in Dohuk (TM), who acted as the clinical supervisor throughout the study, they conducted a refresher training for the 11 CMHWs who were part of the evaluation study in the Dohuk region. This training presented a much-shortened version of the original HAI program that was specific to survivors of torture and imprisonment. CMHWs were presented with an array of 9 techniques to use in counseling clients; for each technique, the CMHWs were taught 4 to 6 activities (Table 1). These activities were to be used according to the individual needs of each client. The refresher training also emphasized core clinical skills of empathic reflection, building trust, emotional expression and regulation, and the conveying of hope and meaning.

The intervention was designed to include 6–12 sessions for each client depending on the client’s needs.

**TABLE 1:** HAI Refresher Training Techniques and Activities for CMHWs

Techniques	Activities
Psychoeducation	Give clients, families, or communities information on psychological problems.
	Reduce stigma about problems and treatment.
	Teach how thoughts, behaviors, and feelings can influence each other positively.
	Explain how talk therapy can help.
Treatment planning	Make arrangements with the client to begin treatment (e.g., confidentiality).
	Agree on how to continue treatment (e.g., weekly sessions, involving family if needed).
	Explain the way treatment will end.
	Describe follow-up assistance if needed after sessions end.
Empowerment	Help clients develop skills and use positive actions and attitudes.
	Start with small changes and help them focus on better parts of life, not only problems.
	Grow from a view of themselves as dependent to better able to care for themselves.
	Reduce feelings of helplessness by being more active and involved with family and community.
Motivation	Encourage clients to come to treatment regularly and make recommended changes in their behavior and thinking.
	Normalize their problems.
	Emphasize the progress they are making.
	Use the treatment relationship for emotional support with empathic listening and reflective techniques.
Crisis management	Assess for suicide or self-injury.
	Use safety plan if needed.
	Be more directive if needed.
	Involve family or other resources if needed.
	Get more consultation and supervision if needed.
	Change the balance between strengths and supports vs. stresses to manage the crisis.
Medication management	Explain how drug therapy can combine with talk therapy to help reduce negative feelings and improve sleep and other problems.
	Advise against the use of alcohol or illegal drugs, which can worsen problems.
	Consult with the physician about a combined therapy plan.
	Monitor for side effects and encourage daily use for later improvement.
Strength building	Identify the skills clients already have.
	Remind them how they have solved problems before.
	Find new ways to feel better, like talking about what is inside.
	Express concern for the negative parts of the client’s life but focus more on the positive (e.g., love of God or their children).
	Emphasize client’s ways of taking care of themselves (e.g., time with friends).
Stress reduction	Assess and encourage client’s interests in positive activities (e.g., praying, exercising).
	Teach relaxation techniques like deep breathing and focusing inside.
	Practice relaxation regularly in counseling and have clients use it at home daily.
	Help clients use relaxation techniques any time they are upset, worried, or cannot sleep.
Advocacy	Identify resources in the family or community that can be used for additional client support.
	Help the client get additional needed services (e.g., medical or legal assistance).
	Promote human rights with equal protection, respect, and benefits for everyone.
	Try to end domestic abuse or child abuse and gender-based violence.
	Connect with other government offices, community programs, and NGOs to increase public awareness about mental health problems and find solutions.

Abbreviations: CMHW, community mental health worker; HAI, Heartland Alliance International.

CMHWs were trained to organize interactions with clients into (1) a preparatory first session that set the stage for the development of a trusting relationship and engaged the client in the work; (2) a series of 4 to 10 “response” sessions in which difficulties related to the principal concerns of PTSD, depression, anxiety, traumatic grief, and impaired functioning were assessed and strategies were taught to address them; and (3) a concluding session that focused on exploring progress made in treatment, consolidation of work and skills learned, and planning for the future. The counseling process was expected to require 6–12 sessions depending on the presenting problems and progress of the client. For counseling to be considered completed, 3 criteria must have been met:

- The participant attended at least 6 sessions.
- The participant expressed no longer feeling the need to attend counseling.
- The CMHW agreed the participant no longer required counseling as evaluated by a review of whether the participant was no longer experiencing many symptoms.

Standards of professional practice were incorporated into the original training as well as into the refresher training prior to trial initiation. These included the need for completing accurate treatment monitoring records, having regular supervision meetings, adherence to ethical standards (e.g., do no harm, maintain professional relations and boundaries), and reporting problems to the supervisor. The CMHWs were advised to monitor their own feelings in the counseling process, follow effective self-care strategies, make use of professional consultation and other support, and maintain a working balance between their clinical experience and their personal lives. These advisements were made to try to avoid CMHWs developing secondary trauma, becoming overly involved in or distanced from the client’s torture experience (enmeshment or detachment), or otherwise burning out.

### Supervision and Monitoring

Fidelity to the treatment model was promoted by monthly on-site group supervision by a psychiatrist (TM) as well as weekly check-ins via mobile phone. If CMHWs had questions during the week, they could contact TM directly via phone. To monitor adherence to the counseling protocol during the on-site meetings, TM reviewed clinical notes, which included how the CMHW responded to the client’s needs and checklists of the different activities the CMHW could have provided. The client monitoring form also included a brief checklist of common mental health symptoms that was used to review client progress and help the CMHW and supervisor decide, together with the client, when treatment would be completed.

The community mental health workers were supervised monthly on-site and weekly via mobile phone.

## METHODS

The study sample from Dohuk governorate was originally planned to be part of a larger trial evaluating 3 different mental health interventions (including the one described in this article) across 3 different regions of Kurdistan, Iraq. The study sample size was calculated for all 3 intervention groups and control participants combined. However, prior to initiating the trials, it became clear that there were substantial population-based differences across the regions, with the population in the Dohuk governorate speaking a different Kurdish dialect, tending toward greater religiosity and political conservativeness, and experiencing different types of trauma exposure given proximity to the Turkish border compared with the other study governorates. We thus made the decision to separate the trials into one trial focusing on 2 of the interventions in the Erbil and Sulaimaniyah governorates^26^ and one trial in Dohuk concentrating only on the supportive counseling program.

### Study Design

This randomized controlled trial was conducted through the primary health clinics staffed by the study CMHWs. Potential trial participants were identified through referral by doctors in the clinics and by referrals from former prisoner organizations. All adults (ages 18 or older) referred to the CMHWs were administered the study instrument as part of the standard intake process for the HAI mental health services. This screening interview also served as the baseline assessment for eligible participants. Trial eligibility criteria comprised:

- Being 18 years or older
- Residing in the Dohuk governorate
- Reporting experiences of torture
- Presenting with significant depressive symptoms
- Not being currently psychotic or actively suicidal
- Being mentally competent to give consent

Experiencing torture was defined as personally experiencing or witnessing physical torture, imprisonment, and/or military attacks. Significant depression was defined as reporting a total score of at least 20 on the 20-symptom, adapted Hopkins Symptom Checklist (HSCL) depression scale and meeting both of the following specific criteria necessary for a DSM-IV (*Diagnostic and Statistical Manual of Mental Disorders*, 4th edition)^27^ diagnosis of a major depressive episode: crying or feeling depressed most or all of the time in the last 2 weeks, and loss of interest in sex or loss of interest in things generally (as evidenced by being unable to enjoy festivals and celebrations most or all of the time) in the last 2 weeks.

If eligible, CMHWs read the study consent form that explained if a person agreed to participate they would be randomly assigned to receive the counseling service beginning immediately or to wait approximately 3 to 5 months before receiving treatment. Persons who did not meet criteria or refused to be in the study were still able to receive services provided by the CMHW but were not included in the trial. Supervisors reviewed all completed assessments and contacted all eligible clients who declined to join the study to confirm their refusal to participate.

Random allocation of participants to intervention or waitlist control was done at the participant level. Study CMHWs were provided with a set of prenumbered consent forms with the designation of intervention or waitlist control status on a piece of paper that was folded and stapled to the back. ID numbers were randomly allocated to study condition by study author (JB) using Stata’s randomization function with a ratio of 3 intervention participants to 1 waitlist control participant, based on the original study design that included all of Kurdistan.

The study was approved by the Johns Hopkins Bloomberg School of Public Health Internal Review Board and by the Ethical Committee of the College of Medicine at the University of Sulaimani in Kurdistan, Northern Iraq.

### Mental Health and Functioning Assessments

A mental health assessment instrument was developed based on initial qualitative data collected in 2008 in the Suleimaniyah governorate of Kurdistan with women and men who had been subjected to torture and/or prison during Saddam Hussein’s regime.^20^ Symptoms of mental distress described by study respondents included feeling sad and depressed, ruminating on the past, loneliness, being withdrawn, fear, anger, anxiety, insomnia, remembering past traumatic events, and avoiding reminders of the past. Based on these results, we selected the following mental health measures to be adapted for the trial:

- The Hopkins Symptom Checklist-25 (HSCL-25^28^^,^^29^ (a 25-item version of the HSCL) for symptoms of depression and anxiety
- The Harvard Trauma Questionnaire (HTQ)^30^ for symptoms of post-traumatic stress
- The Inventory of Traumatic Grief^31^ for symptoms of traumatic grief

We adapted and validated these instruments for the local context using methods described elsewhere.^32^ The adaptation process included adding 22 locally relevant symptoms identified during the previous qualitative study,^20^ including 19 that described features of depression, anxiety, trauma, or traumatic grief but were not already represented on these measures. Of these items, 5 were added to the depression measure, 7 to the post-traumatic stress measure, 1 to the traumatic grief measure, and 3 to the assessment but not a particular symptom scale. In total, we included 70 items in the full mental health instrument (see Bolton et al., 2014^26^ for a list of all mental health symptom items). For all items, participants rated how frequently they experienced each symptom in the prior 2 weeks using an ordinal scale of 0 (never) to 3 (always).

Functionality was defined based on a series of tasks and activities, identified during a prior qualitative study,^32^ regularly done by adults in Dohuk to take care of themselves and their families, and to participate in their community. We developed separate measures for men and women. Respondents were asked to report how much difficulty they had completing each task and activity. Dysfunction for each activity was rated on a Likert scale ranging from 0 (no more difficulty than most other men/women of the same age) to 4 (frequently unable).

We conducted a brief validation study of the mental health and measure of dysfunction, which is described elsewhere in detail.^26^ Briefly, Cronbach’s alpha scores for mental health and dysfunction scales ranged from 0.73 to 0.93, indicating adequate to good internal reliability. Pearson correlation coefficients for combined inter-rater/test-retest reliability (repeat by different interviewer) ranged between 0.73 and 0.86. Intraclass correlation coefficients (ICC) similarly ranged from 0.80 to 0.87.

We compared mean scores for the mental health measures between individuals identified as having each syndrome with those identified as not having the syndromes to evaluate whether our measures could adequately discriminate between them. Discriminant validity was identified for all mental health syndromes in men but only for PTSD in women. Because of the generally good performance of the symptom and dysfunction scales on other tests of validity and reliability for both sexes, we suspected that the poor validity among women was due to testing methods in which we relied on husbands to report the existence of problems among their wives, which may have resulted in misidentification due to husbands being less skilled at assessing the occurrence of these problems among their wives. We therefore concluded that the symptom measures were likely adequate for use among both men and women in this population.

### Waitlist Control Condition

CMHWs contacted waitlist control participants monthly, usually by telephone, for a brief check if they were experiencing substantially greater distress or had become a danger to themselves or others. Control participants were also instructed to contact the CMWHs at any time during the study if their symptoms worsened for assessment and referral if necessary, including transportation to a psychiatrist or the Trauma Rehabilitation and Training Center in Suleimaniyah.

### Post-Intervention Assessment

We aimed to readminister the study instrument within 1 month of completing counseling for the intervention participants and the equivalent (between 3 to 5 months after baseline) for the waitlist controls. The majority (82%, n = 154) of the follow-up interviews were implemented by CMHWs who were blinded to the participant’s treatment status, whereas 18% (n = 34) were implemented by CMHWs or study supervisors who were unblinded. This latter group included persons who dropped out of the trial but were subsequently located by a member of the research team who confirmed that they did not want to continue or be seen again. Rather than risk losing them to follow-up, the research supervisors and staff (instead of CMHWs) did these follow-up interviews despite the lack of blinding. Analyses were done with and without the 34 participants who were assessed unblinded to evaluate the impact of the unblinded subjects.

Trial recruitment ran from June 2009 through June 2010. Due to logistic challenges and difficulty keeping track of study participants (i.e., many often turned off their phones or left the area for work), the average time between baseline and follow-up assessments was 6.4 months for waitlist control participants (range, 3.1 to 10.7) and 5.9 months for intervention participants (range, 2.8 to 13.1). Although this difference was not statistically significant we did control for length of time between assessments in the analyses.

### Sample Size

We calculated the sample size required to detect a 20% greater reduction in depression symptom scores among intervention participants compared with controls, with 80% power and an alpha of 0.05. The choice of 20% represents our estimate of a likely meaningful change. This yielded a sample size of 85 per arm. Estimating a dropout rate of 25%, we increased the recruitment target to 106 in each arm. The 106 participants in the waitlist control condition were to be spread across the 3 intervention programs (i.e., about 26 per treatment program) in the original study design, but because of the decision to evaluate the Dohuk study separately from the other 2 interventions, we continued to enroll participants using the same allocation ratio until our control group reached 50 participants to increase our statistical power. A post-hoc power analysis comparing mean outcomes at follow-up with unequal sample sizes at alpha of 0.05 resulted in 52% power for the depression symptoms outcome and 88% for the dysfunction outcome.

### Statistical Analysis

For each study participant, average baseline and follow-up syndrome-specific and dysfunction scale scores were generated. Our main intent-to-treat analyses used multilevel models with a robust variance estimator and 2 random effects: participant and CMHW. Assessment of treatment effects was based on comparing differences by study arm in the change in average syndrome-specific and dysfunction scale scores from baseline to follow-up (interaction of 2 fixed effects: study arm and time). We controlled for age, sex, marital status (currently married vs. not married), and employment status (any current work vs. not working) based on theory and literature indicating these factors may be potential confounders. We also included variables that differed between treatment and control at baseline or predicted change in outcome as covariates: number of children, disability (yes or no), and length of time between assessments.

Multiple imputation by chained equations was used to account for item-level missingness in scales for any participant as well as missing post-assessment scale scores, employment status, disability status, and length between assessments for those lost to follow-up.^33^ Marital status, years of education completed, and number of children were carried forward for those lost to follow-up. Multiple imputation was also used to estimate missing scale scores for 2 participants whose baseline questionnaires were lost. For these 2 participants, demographic information was carried back from follow-up. All analyses were conducted using Stata version 12.0.^34^

## RESULTS

### Participant Flow Including Losses and Exclusions

A total of 295 adults living in the Dohuk governorate seeking services from a study CMHW were screened for eligibility. CMHWs found 82 participants to be ineligible, and 4 others refused participation (Figure). A total of 209 men and women were randomized to either the intervention (n = 159) or a waitlist control condition (n = 50). Of the 159 allocated to the counseling intervention, 5 never initiated counseling. Of these 5, 3 dropped out of the trial stating they desired financial assistance, 1 was assigned to a CMHW who quit due to increased administrative responsibilities in the hospital, and 1 failed to initiate counseling for an unknown reason but remained under follow-up.

**FIGURE:**
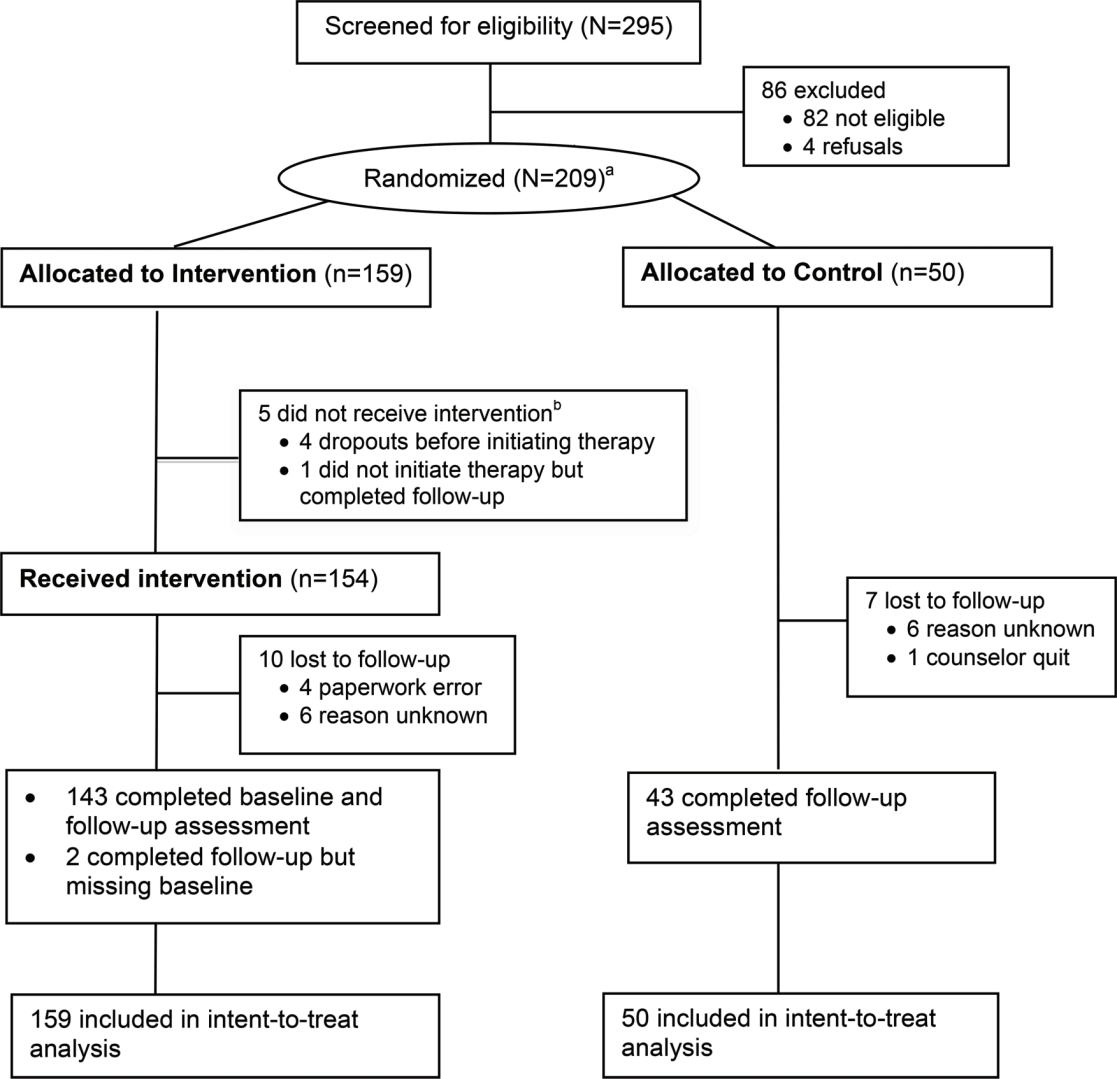
Flow Chart of Study Participants ^a^295 individuals were screened at baseline and 209 randomized. However, data at baseline were missing for 2 individuals randomized to the intervention and followed‐up; thus, we had data for only 293 people screened and 207 randomized. ^b^ Of the 5 people allocated to the study intervention who did not receive it, 3 opted for financial support, 1 was assigned to a counselor who quit, and the last person’s reasons were unknown.

During the course of the trial, 7 (14.0%) individuals were lost to follow-up in the control arm, and 10 (6.3%) individuals who initiated therapy were lost to follow-up in the intervention arm. In total, 188 individuals completed follow-up (90.0%). Individuals lost to follow-up were significantly more likely to be female (*P* = .04), self-employed (*P* = .007), and unmarried (*P* = .04).

### Treatment Completion and Content

Among those who initiated treatment (n = 154), the rate of completion was 95.5% (n = 147). (Some participants completed treatment by definition but did not complete a follow-up assessment.) The mean number of sessions attended was 11.29 (range, 7 to 12) among completers and 1.86 (range, 0 to 3) among non-completers who initiated treatment. Neither treatment completion nor mean number of sessions attended differed significantly by participant sex. Among those who initiated treatment, a higher average baseline depression scale score (*P* = .04) and higher average baseline post-traumatic stress symptoms (*P* = .03) were both related to treatment completion. There was also variation across CMHWs in treatment completion among their clients (*P* = .01); however, all but 3 CMHWs had greater than 90% completion rates among their clients who initiated treatment.

Based on CMHW monitoring forms and supervisory reports, the activities most commonly used by CMHWs included relaxation and psychoeducation on mental health symptoms, treatment, and prognosis. If a client was comfortable with their family having knowledge of their problems, CMHWs visited their families to provide psychoeducation. In 5 instances, CMHWs helped clients find employment. The study intervention emphasized the importance of social support, and CMHWs commonly encouraged clients to engage in social activities (e.g., going to coffee, shopping, or on a picnic) and even accompanied them in some cases.

The community mental health workers most commonly used relaxation and psychoeducation techniques with their clients.

### Baseline Characteristics

Table 2 presents the demographic characteristics of the supportive counseling and waitlist control participants. The average study sample age was 40 years and ranged from 18 to 82. About a third of the sample was female, and about 20% self-reported disability. The majority of the sample was married, approximately half reported being unemployed, and more than 40% reported no education. Demographic characteristics of the participants across the 2 arms were comparable with no differences reaching statistical significance.

**TABLE 2:** Baseline Characteristics of Intent-to-Treat Sample, Dohuk Governorate, Kurdistan, June 2009–June 2010 (N = 207)

	Counseling Intervention (n = 157)^a^	Waitlist Control (n = 50)
Age, years, mean (SD)	40.30 (15.3)	40.76 (12.82)
Female, No. (%)	54 (34%)	15 (30%)
No. of children, mean (SD)	4.80 (4.09)	4.86 (3.91)
Disabled, No. (%)	32 (20%)	9 (18%)
Marital status		
Married, No. (%)	116 (74%)	43 (86%)
Single/divorced/widowed, No. (%)	41 (26%)	7 (14%)
Employment		
Not working, No. (%)	87 (55%)	26 (52%)
Regular work, No. (%)	27 (17%)	11 (22%)
Self-employed, No. (%)	23 (15%)	8 (16%)
Irregular work, No. (%)	20 (13%)	5 (10%)
Education		
None, No. (%)	68 (43%)	24 (48%)
Primary, No. (%)	53 (34%)	14 (28%)
Secondary, No. (%)	29 (18%)	11 (22%)
Bachelors/institutional degree or certificate, No. (%)	7 (4%)	1 (2%)

Abbreviation: SD, standard deviation.

a 159 were allocated to the counseling intervention, but 2 participants’ paperwork at baseline was lost.

### Trial Effects

Estimates of treatment effects across the primary outcomes of depression and dysfunction are presented in Table 3. The intervention had a statistically significant and moderate-sized effect on depression symptoms (Cohen’s d, 0.57; *P* = .02) and dysfunction (Cohen’s d, 0.53; *P* = .03). Although the intervention also appeared to decrease symptoms of the secondary outcomes (post-traumatic stress, traumatic grief, and anxiety), these effects were small. Of the secondary outcomes, the effect was statistically significant for anxiety (Cohen’s d, 0.41; *P* = .01) and marginally significant for post-traumatic stress (Cohen’s d, 0.35; *P* = .07) and traumatic grief (Cohen’s d, 0.26; *P* = .08). Exclusion of baseline covariates resulted in slightly smaller effects across outcomes (-0.04 to -0.05 difference in Cohen’s d) but did not meaningfully change results. Analyses conducted removing the 34 participants who were assessed unblinded to their treatment status resulted in smaller effect sizes for depression (Cohen’s d, 0.45; *P* = .12), dysfunction (Cohen’s d, 0.47; *P* = .08), and anxiety (Cohen’s d, 0.36; *P* = .06) and larger effect sizes for trauma (Cohen’s d, 0.43; *P* = .11) and traumatic grief (Cohen’s d, 0.28; *P* = .11).

The intervention had a statistically significant and moderate-sized effect on depression and dysfunction.

**TABLE 3:** Adjusted Treatment Effects on Primary and Secondary Study Outcomes,^a^ Dohuk Governorate, Kurdistan, June 2009–June 2010 (N = 209)

	Counseling Intervention (n = 159) Score (95% CI)	Waitlist Control (n = 50) Score (95% CI)	Adjusted Net Effect Score (95% CI)	Effect Size Estimate^b^	*P* Value
**Primary Outcomes**					
Depression					
Baseline	1.61 (1.51, 1.71)	1.59 (1.44, 1.74)			
Follow-up	0.78 (0.67, 0.89)	0.97 (0.75, 1.20)			
Pre-post change	-0.83 (-0.98, -0.69)	-0.62 (-0.86, -0.37)	-0.22 (-0.39, -0.04)	0.57	.02
Functional impairment					
Baseline	1.92 (1.69, 2.15)	1.86 (1.56, 2.16)			
Follow-up	1.16 (0.95, 1.38)	1.49 (1.15, 1.83)			
Pre-post change	-0.76 (-1.06, -0.45)	-0.37 (-0.83, 0.09)	-0.39 (-0.74, -0.03)	0.53	.03
**Secondary Outcomes**					
Post-traumatic stress					
Baseline	1.34 (1.22, 1.46)	1.35 (1.17, 1.52)			
Follow-up	0.73 (0.64, 0.83)	0.86 (0.69, 1.04)			
Pre-post change	-0.61 (-0.74, -0.48)	-0.48 (-0.68, -0.29)	-0.13 (-0.27, 0.01)	0.35	.07
Anxiety					
Baseline	1.30 (1.17, 1.43)	1.25 (1.08, 1.41)			
Follow-up	0.66 (0.53, 0.80)	0.81 (0.59, 1.03)			
Pre-post change	-0.64 (-0.83, -0.44)	-0.44 (-0.67, -0.21)	-0.19 (-0.35, -0.04)	0.41	.01
Traumatic grief					
Baseline	0.87 (0.74, 1.01)	0.86 (0.68, 1.03)			
Follow-up	0.38 (0.31, 0.44)	0.47 (0.32, 0.62)			
Pre-post change	-0.50 (-0.59, -0.40)	-0.38 (-0.50, -0.27)	-0.11 (-0.24, 0.02)	0.26	.08

Abbreviation: CI, confidence interval.

a Model-estimated differences after adjusting for age, sex, employment status, time between assessments, number of children, and marital status in all models. All models include multiple imputation by chained equations for missing data and for missing outcomes due to loss to follow-up. Robust standard error estimators are used to account for clustering by counselor.

b Measured using Cohen’s *d* statistic and pooled baseline variances.

## DISCUSSION

We assessed the impact of a trauma-informed support, skills, and psychoeducation intervention on depression symptoms and dysfunction scores among survivors of torture and related traumatic experiences living in the Dohuk governorate of Kurdistan. We found moderate effect sizes for the study intervention on the primary outcomes, which is consistent with the 16 studies of mental health treatments for survivors of torture and trauma that reported effect sizes included in the review by McFarlane and Kaplan.^35^ Treatment effects on the secondary outcomes of post-traumatic stress, anxiety, and traumatic grief were smaller, with the study intervention having the greatest impact on anxiety and the smallest impact on traumatic grief.

These findings are consistent with what we would expect to see from a trauma-informed approach that emphasizes building a sense of internal safety and regulation by coping with and managing reactions to symptoms. In a progressive treatment model, this type of work is characterized as setting the stage for later, more in-depth narrative work that focuses on addressing underlying trauma experiences and associated PTSD symptoms, which the HAI intervention did not include.^8^^,^^29^^,^^30^

Similar to other randomized controlled trials of psychological interventions in low- and middle-income countries,^7^^,^^8^^,^^26^ we observed average symptom improvement among the control sample. Study eligibility was based in part on having severe enough symptoms to warrant a mental health intervention. Given that mental health symptoms and accompanying dysfunction can vary over time, average improvement over time may be due to regression to a population mean. Improvement may also be a result of participants experiencing some relief in symptoms because an interviewer is taking the time to ask them about their problems. It is also possible that control participants accessed other supportive services in the community unrelated to the clinical services offered. Regardless, a study without a control sample would have likely overestimated the intervention effectiveness.

Given the nature of the intervention and the context of historical and ongoing trauma exposure, the reductions in depression symptoms and improvements in functioning seem particularly notable. While depression manifests in culturally distinct ways, a core feature typically includes negative beliefs about oneself and one’s life. It seems plausible that building skills in symptom identification, stress reduction, and emotion regulation resulted in a greater sense of self-efficacy for participants and reductions in depression symptoms. The HAI intervention also emphasized the centrality of the therapeutic alliance in trauma-informed work, one that is characterized by compassion, positive regard, and fostering a sense of hope. Although not specific to the Iraqi context, a strong client-provider relationship is emphasized as a core therapeutic factor across many different treatment approaches and has been found to be an important element in predicting positive client outcomes.^31^ While there is good conceptual rationale for these ideas, future studies looking at these particular intervention elements—self-efficacy, therapeutic alliance—and client outcomes are needed to test these hypotheses.

A strong client-provider relationship has been found to be an important element in predicting positive client outcomes.

The results of this study are particularly relevant as many organizations working in humanitarian contexts are moving forward with the development and implementation of what are commonly referred to as “low-intensity interventions,” i.e., interventions delivered and/or supported by community-based providers without formal mental health training and provided with limited support by formal health care institutions. These interventions fit within the larger WHO model for global mental health services – the Mental Health Gap Action Programme (mhGAP).^36^ Like the HAI intervention evaluated in this study, these low-intensity interventions tend to be based on the techniques used in cognitive behavioral therapy (CBT) that support relieving distress and improving daily functioning.^37^ For example, the World Health Organization (WHO) has developed such an intervention, Problem Management Plus (PM+), that, similar to the HAI program, is aimed at treating a wide range of presentations of distress. The PM+ intervention integrates problem-solving and behavioral techniques in a program that can be implemented by community providers with ongoing supervision.^38^ Ongoing trials of these low-intensity, supportive counseling programs will be important to understand their effectiveness in reducing the burden of mental health problems among trauma-affected populations.

Our choice to use depression symptoms as criteria for entry into this trial was based on our previous qualitative study that found that depression-like symptoms were described most frequently in discussions of mental distress with survivors of torture in Kurdistan.^20^ This is in contrast to other mental health intervention studies for torture survivors that have generally included only those suffering from PTSD-related symptoms.^35^ The choice to use depression symptoms for screening was to identify those survivors who expressed a high degree of distress with respect to a group of symptoms that were clearly important locally (depression).

While depression symptoms were the main entry criteria, the study intervention was originally designed to assist survivors with a wide range of mental and psychosocial issues. Therefore, our finding of small to moderate effects across multiple mental health outcomes suggests that the intervention produced a wide-ranging, although limited, effect on these specific outcomes. The moderate reduction in dysfunction may reflect the cumulative impact of reduction in the symptoms we measured as well as impacts on other problems that we did not assess. The moderate impacts, while significant, do not approach the effect sizes seen when evidence-base psychotherapy interventions have been implemented for the same outcomes with similar populations in Iraq,^2^^,^^32^ suggesting that for organizations that want to specifically target common mental disorders, evidence-based treatment can have more impact for similar levels of training and supervision resources.

### Limitations

Several limitations must be acknowledged. As part of the informed consent process, the waitlist control group was informed that they would be offered treatment regardless of their scores on the follow-up interview to reduce the possibility that they might try to report severe symptoms. It is still possible, however, that controls may have seen an incentive to report more distress at follow-up than intervention participants. In addition, as controls did not meet with CMHWs routinely, we are uncertain as to how much of the intervention’s impact was due to counseling content as opposed to the act of meeting weekly with CMHWs. We also did not explore the cultural understanding of what counseling might mean in this community and how the experience of counseling may differ based on participant sex or other cultural factors. Another limitation is that the participants were chosen on the basis of significant levels of depressive symptoms. The HAI program was originally designed to be a referral program for clients with a wider range of presenting problems, so the results may not represent the typical target population for this program. Our use of unblinded assessors for approximately 15% of the post-intervention assessments resulted in our interviewing a larger proportion of the study participants but did introduce some bias into our findings; while the general conclusions were not affected, the significance of the findings were somewhat affected. However, it is not clear if the variation in significance was due to unblinding or due to the change in sample size when the unblinded sample was excluded. Finally, we also are unable to assess if the effects seen following counseling were sustained, due to a lack of long-term follow-up.

## CONCLUSION

The study intervention was developed as a trauma-informed treatment approach that could be provided by CMHWs for torture and trauma survivors living in rural areas of Northern Iraq. This intervention was well defined, based on extensive practice experience with torture survivors and trauma-affected communities, and the CMHWs were provided with training on the content of the program as well as when and how to implement the different activities and therapeutic skills. The intervention was adapted by a clinician with experience providing trauma-focused care and the CMHWs received regular clinical supervision from a local psychiatrist. The results suggest that this type of well-supervised, trauma-informed intervention can provide moderate improvement for depression and anxiety symptoms and functional impairments in torture- and trauma-affected communities. The current WHO model—Mental Health Gap Action Programme (mhGAP)^36^—requires that moderate to severe cases of mental health problems be treated via referral to specialist services. Under such a model, supportive services that have a moderate effect and are widely accessible at the community level, such as the one studied here, can play an important role as the first line of treatment for less severe cases. However, it is not clear at this time how good access to specialist services in low- and middle-income countries is to be achieved, leaving community-level services as the only accessible option. Service providers will need to decide whether this type of supportive intervention is sufficient for communities with a high burden of moderate to severe cases of common mental disorders, or whether more effective community-based interventions will also be needed.

Well-supervised supportive counseling can provide moderate improvement for depression and anxiety symptoms and functional impairments in trauma communities.

## Notes

**Peer Reviewed**

**First Published Online:** 2016 Sep 8
